# A Consistent Framework for Coupling Basal Friction With Subglacial Hydrology on Hard‐Bedded Glaciers

**DOI:** 10.1029/2021GL097507

**Published:** 2022-07-08

**Authors:** Adrien Gilbert, Florent Gimbert, Kjetil Thøgersen, Thomas V. Schuler, Andreas Kääb

**Affiliations:** ^1^ University Grenoble‐Alpes CNRS IGE Grenoble France; ^2^ Department of Geosciences University of Oslo Oslo Norway; ^3^ Physics of Geological Processes Department of Physics The Njord Centre University of Oslo Oslo Norway

**Keywords:** glacier sliding, subglacial hydrology, ice dynamics

## Abstract

Below hard‐bedded glaciers, both basal friction and distributed subglacial drainage are thought to be controlled by a network of cavities. Previous coupled hydro‐mechanical models, however, describe cavity‐driven friction and hydraulic transmissivity independently, resulting in a physically inconsistent cavity evolution between the two components of the models. Here, we overcome this issue by describing the hydro‐mechanical system using a common cavity‐evolution description, that governs both transient friction and hydraulic transmissivity. We show that our coupling approach is superior to previous formulations in explaining a unique observation record of glacier sliding speed from the French Alps. We find that, at multi‐day to multi‐decadal timescales, sliding speed can be expressed as a direct function of basal shear stress and water discharge, without accounting for water pressure, which simply adjusts to maintain the cavitation ratio needed to accommodate the water supply.

## Key Points

- We introduce a consistent framework to describe the effect of cavitation on both friction and hydraulic transmissivity
- This approach reduces the number of parameters of the coupled hydro‐mechanical problem and shows better performance in explaining our data
- We find that seasonal sliding variations are well captured assuming cavities in equilibrium with their average discharge

## 1 Introduction

Flow variability of ice sheets and glaciers adds a large uncertainty to projections of their evolution and their future contribution to sea level rise (e.g., Mouginot et al., 2019; Ritz et al., 2015; Shepherd et al., 2019). Ice flow variability arises from the complex relationship between sliding speed, stress balance, water pressure, and temperature at the glacier base, all of which, in addition, depend on the properties of the substrate beneath the ice (Cuffey & Paterson, 2010). In particular, the difficulty in computing basal water pressure (e.g., Downs et al., 2018; Flowers, 2015) limits the predictive power of current ice sheet models (Ritz et al., 2015), and therefore the ability to project the future of the cryosphere under climate change.

Two‐way coupled models of ice flow and subglacial hydrology, in which sliding velocity has an effect on subglacial hydrology and vice‐versa (e.g., Hewitt, 2013; Hoffman & Price, 2014; Pimentel et al., 2010), provide useful tools to test the sensitivity of ice dynamics to melt water supply. These models are also needed to evaluate the subglacial hydrology and friction theories by confronting modeled with observed ice velocities (Brinkerhoff et al., 2021). Ice flow and subglacial hydrology models are usually linked by a friction law that relates water pressure, basal shear stress, and sliding velocity, and an equation linking the sliding speed to the efficiency of the distributed drainage system (e.g., Bueler & van Pelt, 2015; Gagliardini & Werder, 2018; Hoffman & Price, 2014). The distributed subglacial drainage system under hard‐bedded glaciers consists of a network of connected cavities (Iken & Bindschadler, 1986; Kamb, 1987; Walder, 1986) which, in current models, is represented by a macro‐porous sheet (Bueler & van Pelt, 2015; De Fleurian et al., 2014; Flowers, 2015; Hewitt, 2011; Schoof et al., 2012; Werder et al., 2013). It is commonly assumed that the thickness of this water sheet evolves through time according to a competition between the opening of cavities due to sliding over bedrock undulations and their closure by creep deformation (Schoof et al., 2012; Walder, 1986).

Although the water‐sheet thickness is linked to the cavitation process which, in turn, controls the basal friction (Fowler, 1986; Lliboutry, 1968), its evolution is typically considered independent of the friction law (Hewitt, 2013). Previous two‐way‐coupled modeling approaches use two distinct parameterizations to describe the influence of cavities on sliding speed and the water‐sheet thickness (e.g., Brinkerhoff et al., 2021; Bueler & van Pelt, 2015; Gagliardini & Werder, 2018; Hewitt, 2013; Hoffman & Price, 2014; Sommers et al., 2018). This leads to the simultaneous existence of two independent descriptions of the cavity size, and thus to inconsistent coupling. Moreover, sliding laws used in previous approaches neglect rate‐weakening friction (Fowler, 1986; Gagliardini et al., 2007; Helanow et al., 2020; Schoof, 2005) and assume steady‐state cavity behavior, a condition which may not be fulfilled given that the cavity evolution timescale (few days) is expected to be longer than the water pressure variations timescale (few hours).

In this study, we overcome the limitations listed above by developing a framework in which existing theories are coupled together such that the frictional state and the drainage efficiency are both controlled by a consistent transient behavior of cavities. We evaluate the model performance against a unique 28‐year record of sliding speed and water discharge from underneath the Argentière Glacier in the French Alps (Gimbert et al., 2021; Vincent & Moreau, 2016). This data set provides unprecedented constraints on subglacial parameters and allows characterizing the behavior of the hydro‐mechanical system from multi‐day to multi‐decadal time scales.

## 2 Materials and Methods

### 2.1 Unifying the Description of Drainage and Friction

Under a steady‐state situation, cavity geometry is at equilibrium with the sliding velocity *u*_*b*_ and the effective pressure $N=p_{i}-p_{w}$ (where $p_{i}$ and $p_{w}$ denote the ice and water pressure, respectively) such that basal shear stress $\tau_{b}$ is only a function of $u_{b}$ and *N* (Diego et al., 2022; Gagliardini et al., 2007; Schoof, 2005). This is however no longer the case in a transient situation, where cavity geometry does not necessarily have the time to fully adjust to changing sliding velocities and effective pressures. In this case, the friction law is expected to be of the form $\tau_{b}=f\left(u_{b},N,\theta \right)$ (Diego et al., 2022; Iken, 1981), where *θ* is a variable describing the cavity geometry. Although calculations of force balance at the sliding interface suggest that a transient sliding law should incorporate an instantaneous dependency on the effective pressure (Iken, 1981; Schoof, 2005); here, we neglect this aspect (as also done in Thøgersen et al. (2019) and Tsai et al. (2021)) and we use a friction law of the form $\tau_{b}=f\left(u_{b},\theta \right)$. Following Thøgersen et al. (2019), we assume that *θ* is a dimensionless cavitation state that allows the transient friction law to be expressed as

(1) $$\tau_{b}^{m}=\left(1-\theta \right)\frac{u_{b}}{A_{s}},$$

where *A*_*s*_ the Weertman friction coefficient (m yr^−1^ MPa^−*m*^) and *m* an exponent. Note that we modified the original definition of θ by Thøgersen et al. (2019) to obtain θ tending to 0 when no cavitation occurs, and that the formulation in Equation (12), (13) is equivalent to equation 4 in Tsai et al. (2021), although the envisioned underlying physics and scales may differ. The cavitation state can be seen as a state variable in an analogy with the “rate and state” friction (Ruina, 1983), although here *θ* corresponds specifically to the cavitation process. *θ* monotonically increases with any variables describing cavity size (e.g., length or height), and is expected to be related to the water sheet thickness *h* considered in subglacial hydrology models, as described later in this section. As shown more specifically below, the particularity of the friction law in Equation (12), (13), in comparison to other “rate and state” approaches applied in glaciology (Goldberg et al., 2014; Lipovsky & Dunham, 2016; Minchew & Meyer, 2020; Zoet et al., 2021), is that its steady‐state form is equivalent to the friction law established by Gagliardini et al. (2007). In addition to including the current state knowledge on steady‐state, hard‐bed friction, the formulation in Equation (12), (13) is compatible with the experimental findings of Zoet et al. (2021) that a step increase in velocity leads first to a drag increase and then, is followed by a transient decrease due to cavities evolving toward a new steady‐state configuration.

Gagliardini et al. (2007) express steady friction through a single explicit dependency of bed shear stress on effective pressure as

(2) $$\tau_{b}=\mathrm{CN}{\left(\frac{\chi }{1+\alpha \chi^{q}}\right)}^{1/m}\;\mathrm{with}\;\chi =\frac{u_{b}}{C^{m}N^{m}A_{s}}\;\text{and}\;\alpha =\frac{{\left(q-1\right)}^{q-1}}{q^{q}},$$

where *C* is a coefficient describing the maximum shear stress supported by the bedrock and *q* is an exponent. The steady‐state cavitation *θ* ^∗^ (Thøgersen et al., 2019) is obtained by combining Equations 1 and 2:

(3) $$\theta^{*}=1-\frac{1}{1+\alpha \chi^{q}}.$$

We note that $\chi =\frac{q}{1-q}$ at the transition to rate weakening (Gagliardini et al., 2007) and that the steady cavitation state $\theta_{s}^{*}$ at this transition can thus be expressed as

(4) $$\theta_{s}^{*}=\frac{1}{q}.$$

Adapting the formulation introduced by Schoof et al. (2012) for the evolution of the water sheet thickness to impose a steady cavitation state that equals *θ* ^∗^ as defined in Equation (1), (2), we obtain the following formulations for the opening and closing velocities of cavities:

(5) $$v_{open}=\frac{1}{l_{r}}{\left(1-\theta \right)}^{\frac{1}{q}}\;u_{b},$$

(6) $$v_{close}=\frac{1}{l_{r}}\;A_{s}{\left(\frac{\theta }{\alpha }\right)}^{\frac{1}{q}}C^{m}{\left|N\right|}^{m-1}N,$$

where *l*_*r*_ is the mean distance (*m*) between bedrock bumps responsible for the cavitation process and $v_{close/open}$ is the rate at which θ evolves (s^−1^). The temporal evolution of *θ* is thus described by

(7) $$\frac{d\theta }{dt}=\frac{1}{l_{r}}\left(u_{b}{\left(1-\theta \right)}^{\frac{1}{q}}-A_{s}C^{m}{\left|N\right|}^{m-1}N\;{\left(\frac{\theta }{\alpha }\right)}^{\frac{1}{q}}\right).$$

In this study, we use the subglacial hydrological model developed by Werder et al. (2013), which is mainly based on Schoof et al. (2012), with the modification that hydrological transmissivity is determined from the cavitation state θ defined by the friction law (Equation (1), (2)). The water sheet thickness *h* (*m*) and the sheet conductivity are thus expressed through their dependence on the common variable θ. We assume a direct relationship between θ and *h* of the form:

(8) $$h(\theta )=h_{r}\theta^{p_{1}},$$

with *h*_*r*_ the average bedrock bump height (*m*) and *p*_*1*_ an exponent. The evolution equation of *h* becomes:

(9) $$\frac{dh}{dt}=h_{r}p_{1}\theta^{p_{1}-1}\frac{d\theta }{dt},$$

which is qualitatively similar to the one used in previous approaches (e.g., Schoof et al., 2012; Werder et al., 2013), although it involves additional exponents. We define as well the sheet conductivity *k*_*s*_ as a function of θ or *h* as

(10) $$k_{s}\left(\theta \right)=k_{s}^{0}\theta^{p_{2}}=k_{s}^{0}{\left(\frac{h}{h_{r}}\right)}^{p_{2}/p_{1}},$$

where $k_{s}^{0}$ is the intrinsic sheet conductivity and *p*_2_ is an exponent.

### 2.2 Deriving a Discharge‐Driven Sliding Law at Steady‐State

Assuming that the hydraulic potential gradient ∇Φ is constant with time (Shreve's [1972] approximation with constant surface slope and ice thickness gradient), it is possible to derive a steady‐state relationship between subglacial discharge and effective pressure (Delaney et al., 2019; Hewitt & Fowler, 2008; Schoof, 2010; Walder & Fowler, 1994) or cavitation state in our case. This can be used to evaluate sliding velocities without solving the hydro‐mechanical problem.

Following Schoof et al. (2012) combined with Equations 8 and 10 to express the sheet thickness and conductivity as a function of θ, the discharge ***Q***_***s***_ in the cavity network (m^2^ s^−1^) can be expressed as a function of θ as:

(11) $$Q_{s}=k_{s}^{0}\theta^{p_{2}}{\left(h_{r}\theta^{p_{1}}\right)}^{\alpha_{s}}{\left\|\nabla \Phi \right\|}^{\beta_{s}-2}\nabla \Phi ,$$

where Φ is the hydraulic potential (MPa), (*α*_*s*_, *β*_*s*_) are constant exponents and bold variables are vectors. Combining Equation (1), (2) with Equation (1), (2), we obtain the following sliding law as a function of water discharge:

(12) $$\frac{u_{b}}{\tau_{b}^{m}A_{s}}=1+\frac{{\left\|Q_{s}\right\|}^{1/\left(p_{1}\;\alpha_{s}+p_{2}\right)}}{{\left\|Q_{s}^{max}\right\|}^{1/\left(p_{1}\;\alpha_{s}+p_{2}\right)}-{\left\|Q_{s}\right\|}^{1/\left(p_{1}\;\alpha_{s}+p_{2}\right)}},$$

where $Q_{s}^{max}=k_{s}^{0}h_{r}^{\alpha_{s}}{\left\|\nabla \Phi \right\|}^{\beta_{s}-1}$. This sliding law can be viewed as a Weertman‐type law $A_{s}^{eq}\tau_{b}^{m}=u_{b}$ for which the friction coefficient $A_{s}^{eq}$ is a function of water discharge. We note that this approximation is expected to hold only over time spans that are long enough for the cavities to adjust and that discharge should be averaged over that same time spans (one to few days).

Instead of using our modified Equations 1 and 11, a steady‐state expression can be derived from formulations by Schoof et al. (2012) and Gagliardini et al. (2007) (Equation (1), (11)) by combining the relationship given by a steady‐state water sheet thickness, the friction law and the sheet discharge (see Supporting Information). We obtain the following relationship:

(13) $$\frac{u_{b}}{\tau_{b}^{m}As}=1+\alpha {\left(\frac{l_{r}\tilde{A}}{C^{m}A_{s}}\frac{{\left\|Q_{s}\right\|}^{1/\alpha_{s}}}{{\left\|Q_{s}^{max}\right\|}^{1/\alpha_{s}}-{\left\|Q_{s}\right\|}^{1/\alpha_{s}}}\right)}^{q},$$

where $Q_{s}^{max}=k_{s}h_{r}^{\alpha_{s}}{\left\|\nabla \Phi \right\|}^{\beta_{s}-1}$ and $\tilde{A}$ is a constant setting the characteristic time of cavity closure. Both Equations 12 and 13 are in fact equivalent to the steady‐state friction law (Equation 2) but use a different estimation of the effective pressure based on two different evolution equations of the sheet thickness. Equation (1), (11) differs from Equation (1), (11) in the way the power exponent on $Q_{s}$ is linked to $\alpha_{s}$. Implications on the value of $\alpha_{s}$ and the description of hydrologic transmissivity in models are discussed in the results section.

### 2.3 Numerical Model

We implement the coupled problem of ice flow and subglacial hydrology in the finite element tool Elmer/Ice (Gagliardini et al., 2013) which solves for both ice flow and hydrology models. The Stokes equation describing ice flow is solved together with the subglacial hydrology problem following the previous implementation of GlaDS (Werder et al., 2013) in Elmer/Ice (Gagliardini & Werder, 2018), in which we implemented the framework described in Section 2.1. The model is detailed in Supporting Information and all the variables and parameters are summarized in Tables S1 and S2 in Supporting Information S1.

### 2.4 Application to Argentière Glacier, French Alps

We take advantage of the unique simultaneous records of sliding speed, subglacial discharge, and ice thickness available from the Argentière Glacier (Mont Blanc Range, France) to evaluate the performance of our approach and quantify the unknown parameters. The topography of the Argentière Glacier has been continuously monitored since 1975 (Vincent et al., 2009) and the existence of subglacial infrastructure allows a continuous measurement of both subglacial discharge and glacier sliding speed (1990–2020) directly at the glacier base (Gimbert et al., 2021; Vincent & Moreau, 2016). The sliding speed is measured using an instrument called the “cavitometer,” which consists of a fixed wheel that rolls as the ice roof of a natural cavity slides above it. The sliding speed is measured with an accuracy of about 1 cm/day (Vincent & Moreau, 2016). Discharge and sliding speed have been recorded at a daily resolution until 2018, when the previous analog logging system was replaced by a digital solution, and the recording interval has been changed to 30 min.

To focus specifically on the interaction between sliding and hydrology and to limit contributions from other factors such as bed topography, we solve the numerical problem on a glacier with a simplified geometry, represented by a 0.6 × 5 km^2^ ice slab over a bedrock with uniform 6° slope representing the average situation of the Argentière Glacier. The surface topography is constructed using the “plastic approximation” from a given driving stress (Cuffey & Paterson, 2010) and evolves at a daily time scale following the reconstructed basal shear stress at the location of the sliding speed measurements (Gimbert et al., 2021) (see Text S1 and Figure S1 in Supporting Information S1). This methodology allows to impose basal shear stresses as expected in the real setup even though the modeled geometry is simplified.

## 3 Results

### 3.1 Steady‐State Cavitation

**Figure 1:**
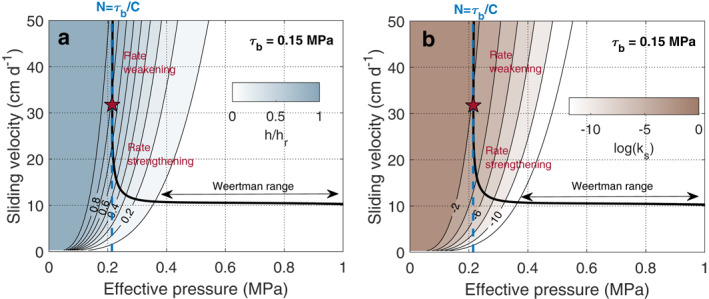
Friction law and hydrological properties of the water‐sheet‐like drainage system at steady state as a function of sliding velocity and effective pressure determined from Equations 3, 8, and 10. (a) Normalized thickness of the water sheet h underneath a glacier (contour lines and associated colored areas) and (b) hydraulic conductivity $k_{s}$. The steady‐state friction law (Equation (3), (8), (10)) for basal shear stresses $\tau_{b}=0.15$ MPa is shown as black bold lines, with red stars showing the transition between rate‐strengthening and rate‐weakening. See Table S1 in Supporting Information S1 for parameter definitions and values.

**Figure 2:**
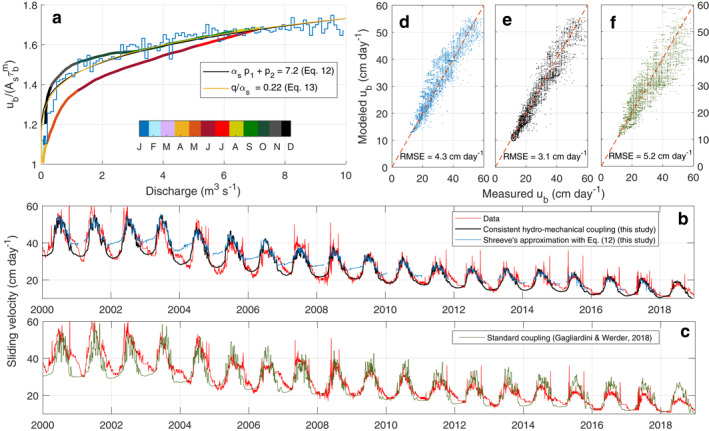
Modeled and observed sliding velocity at Argentière Glacier, France. (a) Observed mean sliding velocity (normalized by $A_{s}\tau_{b}^{m}$) for linearly spaced observed daily mean water discharge bins over the whole time series (blue line) and split by month (colored line). The black and yellow lines show predicted sliding velocity using Shreve's (1972) approximation and Equations 12 and 14, respectively. (b) Observed daily sliding velocities (orange line) compared to modeled sliding velocities using Shreve's (1972) approximation and Equation (3), (8), (10) (blue line) and using the newly introduced consistent hydro‐mechanical coupling (black line). (c) Same as (b) but compared to modeled sliding velocities using a standard coupling (Gagliardini & Werder, 2018) (green line). (d–f) Modeled as a function of measured daily velocities for the period 2000–2018 using Equation (3), (8), (10) (d), the consistent hydo‐mechanical coupling (e), and the standard coupling (f).

The novelty of our study is that the hydraulic transmissivity evolves according to the friction law through its link with the cavitation state θ (Equations 8 and 10). The sheet thickness and the hydraulic conductivity thus increase only when cavitation is affecting the sliding velocity (see Figure 1 for the steady‐state case $\theta =\theta^{*}$). In previous approaches (e.g., Bueler & van Pelt, 2015; Gagliardini & Werder, 2018; Hewitt, 2013; Hoffman & Price, 2014; Pimentel et al., 2010), independent parametrization of the friction law and the water sheet thickness evolution could lead to inconsistent configurations where the water‐sheet thickness is large while cavitation does not occur in the friction law (shown as the “Weertman range” in Figures 1 and 2). The additional constraints given by the coupling reduce the range of effective pressure that allows the water to drain. In particular, it links effective pressure to basal shear stress through imposing averaged effective pressure to be close to $\tau_{b}/C$ for the cavitation to occur and the water drainage to happen (Figures 1a and 1b). The distributed drainage system becomes able to accommodate the melt water supply only in a narrow range of effective pressure which is sensitive to the basal shear stress value.

### 3.2 Evaluating the Discharge‐Driven Sliding Law Using Observations at the Argentière Glacier

Assuming that $Q_{s}$ is proportional to the measured total discharge, Equation (3), (8), (10) provides a relationship between sliding speed and water discharge that can be compared to observations from the Argentière Glacier. Observed mean sliding velocity per given discharge intervals is well predicted by our framework providing that $\alpha_{s}p_{1}+p_{2}$  = 7.2 (Figure 2a). The model also captures the intra‐monthly sliding variations during the melting period (Figures 2b and 2c), confirming that the equilibrium between discharge and the cavitation state is reached over a time scale of a few days (Bartholomaus et al., 2011). This shows that changes in cavitation state accommodate the variability in water supply at multi‐day and seasonal time scales, while changes in the hydraulic potential gradient, which are not accounted for here, likely only accommodate the variability in water supply at subdaily time scale. This supports that, on average, effective pressure and sliding velocity are driven by the cavitation state needed to accommodate water input and that the hydraulic potential gradient can be considered as constant in time. The relationship obtained from previous approaches (Equation (3), (8), (10)) shows similar agreement with data for $\frac{q}{\alpha_{s}}$  = 0.22 (Figure 2a). However, given that *q* ≥ 1, a value of $\alpha_{s}$ ≥ 5 is needed to explain the observations. Such a high value is unlikely, given that $\alpha_{s}$ ≈ 5/4 is appropriate for turbulent flow as described by the Darcy‐Weisbach law and $\alpha_{s}$ ≈ 3 is appropriate for laminar sheet flow (Hewitt, 2013). We attribute the significant non‐linearity observed in the relationship between discharge and sliding velocity to the existence of a threshold cavitation state from which the cavities start to connect (large value of $p_{2}$) rather than a high value of $\alpha_{s}$. This result aligns with direct field observations of permeability acting as a binary switch, between connected and unconnected drainage networks (Andrews et al., 2014; Rada & Schoof, 2018).

### 3.3 Transient Coupled Numerical Model

Solving the full hydro‐mechanical coupled problem including channel dynamics and transient cavitation on an idealized setup allows to study daily to seasonal variations of sliding velocity that can be compared with the Argentière Glacier observations to calibrate unknown hydrological parameters and test the potential effect of channels. Parameter estimations are based on best fit between observed and modeled sliding velocity (see Supporting Information). In particular, this allows us to constrain separately the exponents *p*_*1*_ = 0.6 and *p*_*2*_ = 6.5 (Figure S2a in Supporting Information S1) and highlight the behavior of the cavity system regarding its hydrological properties. The low value of *p*_*1*_ indicates that the cavity volume increases as soon as the cavitation state θ increases while the high value of *p*_*2*_ indicates that the cavity network undergoes an abrupt transition in terms of connectivity as a certain threshold of cavitation state is reached (Figure S2b in Supporting Information S1).

Our coupled model captures remarkably well both the long‐term (multi‐decadal) and short‐term (multi‐day) variations in sliding velocity observed at the Argentière Glacier (Figures 2b and 2e and Figure S3a in Supporting Information S1). The model also accurately reproduces the average characteristics of the observed seasonal variability of sliding speed as a function of discharge (Figures S3b–S3d in Supporting Information S1). The fact that modeled sliding velocities remain close to those estimated using Equation (3), (8), (10) and Shreve's (1972) approximation during the melting period (Figure 2b) supports our suggestion that the system tends to adapt its hydraulic transmissivity through enhanced cavitation rather than by increasing pressure gradient to accommodate the water supply at multi‐days and seasonal timescales.

To compare our predictions with those using other approaches, we performed the same simulation using the standard coupling in GlaDS (Werder et al., 2013) as implemented in Elmer/Ice (Gagliardini & Werder, 2018) where the sheet thickness evolution and the resultant effective pressure are computed independently of the friction law (see Text S4 in Supporting Information S1). Results show that the best match with observations leads to a minimum RMSE of 5.2 cm day^−1^ whereas our approach gives a considerably lower RMSE of 3.1 cm day^−1^ (Figure 2f and Figure S4 in Supporting Information S1). In addition, best results using the standard coupling are obtained for unusual parameter values of $\alpha_{s}\in \left[5,\;7\right]$ and $\tilde{A}\in \left[1.0,\;3.0\right]10^{-22}$ Pa^−3^ s^−1^ which are far from the more realistic values $\alpha_{s}=1.25$ according to the Darcy‐Weisbach law and $\tilde{A}=5.0\;10^{-25}$ Pa^−3^ s^−1^ for temperate ice viscosity. In particular, the multi‐day variability is not well represented due to the water pressure variability being too high as a result of not being well regulated by the response of drainage efficiency to cavitation changes (Figure 2c). Even more importantly, the change in amplitude of the seasonal cycle in response to the multi‐decadal change in ice thickness is not well represented using GlaDS compared to using our present approach (Figure 2c). In both approaches, the cavitation state (our study) and the water sheet thickness (GlaDS) remain similar every summer because both are set by the water discharge. In our approach, this condition translates to the quantity $\frac{u_{b}^{summer}}{A_{s}\tau_{b}^{m}}$ being conserved, such that the ratio between winter and summer velocities varies proportionally to $\tau_{b}^{m}$, consistent with observations (Gimbert et al., 2021). In contrast, the standard coupling fails to reproduce this behavior. With respect to the friction law only, using Equation (3), (8), (10) instead of a more conventional *N*‐dependent friction law (Gagliardini et al., 2007; Schoof, 2005) has the considerable advantage that the momentum balance can be solved regardless of the effective pressure value, since the law is rate strengthening at any given time. This allows to use rate‐weakening friction laws, and to solve the hydrological problem under high water input rates in which water pressure can significantly rise (even at overburden) during a short amount of time. However, at short timescale (subdaily), the data shows an instantaneous response of sliding speed to water pressure. This suggests that friction reacts to change in effective pressure at fixed cavitation ratio and that Equation (3), (8), (10) should include a dependency on effective pressure in order to properly capture subdaily sliding velocity variations.

Figure S3d in Supporting Information S1 highlights a seasonal hysteresis that is captured by our model due to seasonal glacier thickness variation and associated driving stress changes (Gimbert et al., 2021). The normalization of the sliding speed by $A_{s}\tau_{b}^{m}$ removes this hysteresis (Figure 2a) and shows how the efficiency of the drainage at low water pressure in the Argentière Glacier does not increase during the melting season, as would be expected if channelization was occurring. This can be explained by a dominance of drainage through the cavity network which is not influenced by the development of the Röthlisberger‐channel (R‐channel) network. However, the simulation shows that, in winter, when the glacier slides in the Weertman range (no cavitation), the cavitation state becomes insignificant and the transmissivity of the water‐sheet‐like drainage vanishes, making the water to primarily drain through residual R‐channels. We find that these channels are in equilibrium with their discharge and impose a constant effective pressure independent of ice thickness in January/February/March (Figure S6 in Supporting Information S1). This result confirms the suggestion by Gimbert et al. (2021) that effective pressure is constant across winters.

## 4 Discussion and Conclusion

**Figure 3:**
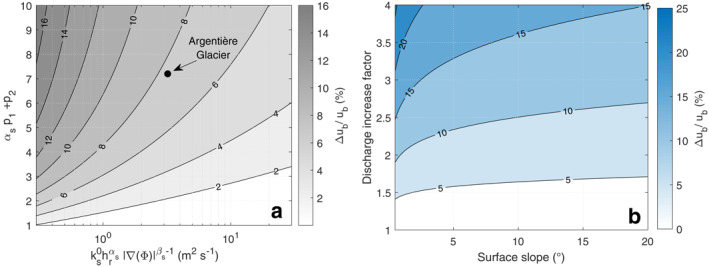
Sliding speed sensitivity to discharge increase determined from Equation (3), (8), (10) using Shreve's (1972) approximation. (a) Sliding speed increase (%) in response to doubling water discharge as a function of the exponent $\alpha_{s}p_{1}+p_{2}$ and the maximum drainage capacity $Q_{s}^{max}=k_{s}^{0}h_{r}^{\alpha_{s}}{\left|\nabla \Phi \right|}^{\beta_{s}-1}$ (see Table S2 in Supporting Information S1 for parameters definition). The black dot indicates the parameter set quantified for Argentière Glacier in this study. (b) Sliding speed increase (%) as a function of surface slope and discharge increase factor for the parameters determined at Argentière Glacier.

In this study, we introduce a missing link between modeling subglacial hydrology and glacier sliding by using the friction law to describe the basal hydraulic transmissivity. We demonstrate that, at the multi‐day time scale, sliding speed is set by the adjustment of the cavitation state needed to accommodate the water supply to the glacier bed. It follows that, at this time scale, water pressure is not a relevant variable to be estimated through complex hydrological models. Rather, melt‐driven subglacial discharge through the cavity system would be a more appropriate variable to predict changes in sliding speed. In the absence of an efficient drainage system, the analytical solution in Equation (3), (8), (10) enables estimating an upper bound for ice‐sheet and glacier acceleration associated with increase in melt water supply into the future. We show that the sensitivity of sliding speed to water discharge is a function of the maximum drainage capacity of the cavity network (m^2^ s^−1^) defined by $Q_{s}^{max}=k_{s}^{0}h_{r}^{\alpha_{s}}{\left|\nabla \Phi \right|}^{\beta_{s}-1}$ and the exponent $\alpha_{s}p_{1}+p_{2}$ (Figure 3a). Assuming that the values of the terms $k_{s}^{0}h_{r}^{\alpha_{s}}$ and $\alpha_{s}p_{1}+p_{2}$ inferred in this study are representative of hard‐bedded glaciers in general and that the hydraulic potential gradient is dominated by the surface slope, we can determine this sensitivity as a function of surface slope only (Figure 3b). For example, given that surface slopes of the Greenland ice sheet are smaller than 2°, a surface runoff increase by a factor of 2–4 expected over the next century (Fettweis et al., 2013) would, according to our findings, lead to a maximum increase in sliding speed of about 10%–25% (Figure 3b).

Interestingly, our findings also show that the equilibrium between drainage efficiency and cavitation state continues to hold even if the required cavitation state lies in the rate weakening range ($\theta_{s}>\frac{1}{q}$) (see Figure S7 in Supporting Information S1). In addition to the existence of this range being questioned for real bed topography (Helanow et al., 2021; Schoof, 2005), our results show that rate‐weakening, even if existing, would not be effective due to the hydrological feedback stabilizing the sliding speed. This is presumably a property of hard‐bedded glaciers where substantial cavitation can occur and the drainage efficiency can significantly increase with sliding speed. This would, in turn, explain the lack of surge observations of hard‐bedded glaciers (Cuffey & Paterson, 2010), such that opening of subglacial cavities would prevent sustaining high water pressure during the surge.

Our findings emphasize that glacier sliding is not only determined by a friction law but also by a “transmissivity law” that needs to be carefully determined and related to the friction state. We believe that the combination of these two laws, including an unstable friction branch, as shown here, is able to explain a wide range of glacier flow behaviors such as seasonal speed up, surge, or catastrophic detachment. Further study should examine how the parameters $p_{1}$, $p_{2}$, $k_{s}^{0}$, and $h_{r}$ rely on bedrock geometry or the presence of sediment (soft bed) and investigate how much they can potentially vary from one place to another. A direct dependency of the transient friction law on effective pressure should also be introduced in order to study short‐term velocity changes due to diurnal surface melt variations, strong rain events, or lake drainages.

## Supporting information

Supporting Information S1

## Data Availability

The modeling code is based on the open‐source code Elmer/Ice available at http://elmerice.elmerfem.org/wiki/doku.php. The data set used in this study is available on the Zenodo platform (https://doi.org/10.5281/zenodo.4286111).

## References

[grl64469-bib-0001] Andrews, L. C. , Catania, G. A. , Hoffman, M. J. , Gulley, J. D. , Luthi, M. P. , Ryser, C. , et al. (2014). Direct observations of evolving subglacial drainage beneath the Greenland Ice Sheet. Nature, 514(7520), 80–83. 10.1038/nature13796 25279921

[grl64469-bib-0002] Bartholomaus, T. C. , Anderson, R. S. , & Anderson, S. P. (2011). Growth and collapse of the distributed subglacial hydrologic system of Kennicott Glacier, Alaska, USA, and its effects on basal motion. Journal of Glaciology, 57(206), 985–1002. 10.3189/002214311798843269

[grl64469-bib-0003] Brinkerhoff, D. , Aschwanden, A. , & Fahnestock, M. (2021). Constraining subglacial processes from surface velocity observations using surrogate‐based Bayesian inference. Journal of Glaciology, 67(263), 385–403. 10.1017/jog.2020.112

[grl64469-bib-0004] Bueler, E. , & van Pelt, W. (2015). Mass‐conserving subglacial hydrology in the Parallel Ice Sheet Model version 0.6. Geoscientific Model Development, 8(6), 1613–1635. 10.5194/gmd-8-1613-2015

[grl64469-bib-0005] Cuffey, K. M. , & Paterson, W. S. B. (2010). The physics of glaciers (4th ed.). Amsterdam: Academic Press.

[grl64469-bib-0006] De Fleurian, B. , Gagliardini, O. , Zwinger, T. , Durand, G. , Le Meur, E. , Mair, D. , & Råback, P. (2014). A double continuum hydrological model for glacier applications. The Cryosphere, 8(1), 137–153. 10.5194/tc-8-137-2014

[grl64469-bib-0007] Delaney, I. , Werder, M. A. , & Farinotti, D. (2019). A numerical model for fluvial transport of subglacial sediment. Journal of Geophysical Research: Earth Surface, 124(8), 2197–2223. 10.1029/2019JF005004

[grl64469-bib-0008] Diego, G. G. d. , Farrell, P. E. , & Hewitt, I. J. (2022). Numerical approximation of viscous contact problems applied to glacial sliding. Journal of Fluid Mechanics, 938. 10.1017/jfm.2022.178

[grl64469-bib-0009] Downs, J. Z. , Johnson, J. V. , Harper, J. T. , Meierbachtol, T. , & Werder, M. A. (2018). Dynamic hydraulic conductivity reconciles mismatch between modeled and observed winter subglacial water pressure. Journal of Geophysical Research: Earth Surface, 123(4), 818–836. 10.1002/2017JF004522

[grl64469-bib-0010] Fettweis, X. , Franco, B. , Tedesco, M. , van Angelen, J. H. , Lenaerts, J. T. M. , van den Broeke, M. R. , & Gallée, H. (2013). Estimating the Greenland ice sheet surface mass balance contribution to future sea level rise using the regional atmospheric climate model MAR. The Cryosphere, 7(2), 469–489. 10.5194/tc-7-469-2013

[grl64469-bib-0011] Flowers, G. E. (2015). Modelling water flow under glaciers and ice sheets. Proceedings of the Royal Society of London: Mathematical, Physical and Engineering Sciences, 471(2176), 20140907. 10.1098/rspa.2014.0907 PMC4991255 27547082

[grl64469-bib-0012] Fowler, A. C. (1986). A sliding law for glaciers of constant viscosity in the presence of subglacial cavitation. Proceedings of the Royal Society of London: Mathematical, Physical and Engineering Sciences, 407(1832), 147–170. 10.1098/rspa.1986.0090

[grl64469-bib-0013] Gagliardini, O. , Cohen, D. , Råback, P. , & Zwinger, T. (2007). Finite‐element modeling of subglacial cavities and related friction law. Journal of Geophysical Research: Earth Surface, 112(F2), F02027. 10.1029/2006JF000576

[grl64469-bib-0014] Gagliardini, O. , & Werder, M. (2018). Influence of increasing surface melt over decadal timescales on land‐terminating Greenland‐type outlet glaciers. Journal of Glaciology, 64(247), 700–710. 10.1017/jog.2018.59

[grl64469-bib-0015] Gagliardini, O. , Zwinger, T. , Gillet‐Chaulet, F. , Durand, G. , Favier, L. , De Fleurian, B. , et al. (2013). Capabilities and performance of Elmer/Ice, a new‐generation ice sheet model. Geoscientific Model Development, 6(4), 1299–1318. 10.5194/gmd-6-1299-2013

[grl64469-bib-0016] Gimbert, F. , Gilbert, A. , Gagliardini, O. , Vincent, C. , & Moreau, L. (2021). Do existing theories explain seasonal to multi‐decadal changes in glacier basal sliding speed? Geophysical Research Letters, 48(15), e2021GL092858. 10.1029/2021GL092858

[grl64469-bib-0017] Goldberg, D. N. , Schoof, C. , & Sergienko, O. V. (2014). Stick‐slip motion of an Antarctic Ice Stream: The effects of viscoelasticity. Journal of Geophysical Research: Earth Surface, 119(7), 1564–1580. 10.1002/2014JF003132

[grl64469-bib-0018] Helanow, C. , Iverson, N. R. , Woodard, J. B. , & Zoet, L. K. (2021). A slip law for hard‐bedded glaciers derived from observed bed topography. Science Advances, 7(20), eabe7798. 10.1126/sciadv.abe7798 33990323 PMC8121427

[grl64469-bib-0019] Helanow, C. , Iverson, N. R. , Zoet, L. K. , & Gagliardini, O. (2020). Sliding relations for glacier slip with cavities over three‐dimensional beds. Geophysical Research Letters, 47(3), e2019GL084924. 10.1029/2019GL084924

[grl64469-bib-0020] Hewitt, I. J. (2011). Modelling distributed and channelized subglacial drainage: The spacing of channels. Journal of Glaciology, 57(202), 302–314. 10.3189/002214311796405951

[grl64469-bib-0021] Hewitt, I. J. (2013). Seasonal changes in ice sheet motion due to melt water lubrication. Earth and Planetary Science Letters, 371(372), 16–25. 10.1016/j.epsl.2013.04.022

[grl64469-bib-0022] Hewitt, I. J. , & Fowler, A. C. (2008). Seasonal waves on glaciers. Hydrological Processes, 22(19), 3919–3930. 10.1002/hyp.7029

[grl64469-bib-0023] Hoffman, M. , & Price, S. (2014). Feedbacks between coupled subglacial hydrology and glacier dynamics. Journal of Geophysical Research: Earth Surface, 119(3), 414–436. 10.1002/2013JF002943

[grl64469-bib-0024] Iken, A. (1981). The effect of the subglacial water pressure on the sliding velocity of a glacier in an idealized numerical model. Journal of Glaciology, 27(97), 407–421. 10.3189/S0022143000011448

[grl64469-bib-0025] Iken, A. , & Bindschadler, R. A. (1986). Combined measurements of subglacial water pressure and surface velocity of Findelengletscher, Switzerland: Conclusions about drainage system and sliding mechanism. Journal of Glaciology, 32(110), 101–119. 10.3189/S0022143000006936

[grl64469-bib-0026] Kamb, B. (1987). Glacier surge mechanism based on linked cavity configuration of the basal water conduit system. Journal of Geophysical Research: Solid Earth, 92(B9), 9083–9100. 10.1029/JB092iB09p09083

[grl64469-bib-0027] Lipovsky, B. P. , & Dunham, E. M. (2016). Tremor during ice‐stream stick slip. The Cryosphere, 10(1), 385–399. 10.5194/tc-10-385-2016

[grl64469-bib-0028] Lliboutry, L. (1968). General theory of subglacial cavitation and sliding of temperate glaciers. Journal of Glaciology, 7(49), 21–58. 10.3189/S0022143000020396

[grl64469-bib-0029] Minchew, B. M. , & Meyer, C. R. (2020). Dilation of subglacial sediment governs incipient surge motion in glaciers with deformable beds. Proceedings of the Royal Society A: Mathematical, Physical & Engineering Sciences, 476(2238), 20200033. 10.1098/rspa.2020.0033 PMC7428031 32821236

[grl64469-bib-0030] Mouginot, J. , Rignot, E. , Bjørk, A. A. , van den Broeke, M. , Millan, R. , Morlighem, M. , et al. (2019). Forty‐six years of Greenland Ice Sheet mass balance from 1972 to 2018. Proceedings of the National Academy of Sciences of the United States of America, 116(19), 9239–9244. 10.1073/pnas.1904242116 31010924 PMC6511040

[grl64469-bib-0031] Pimentel, S. , Flowers, G. E. , & Schoof, C. G. (2010). A hydrologically coupled higher‐order flow‐band model of ice dynamics with a Coulomb friction sliding law. Journal of Geophysical Research: Earth Surface, 115(F4). 10.1029/2009JF001621

[grl64469-bib-0032] Rada, C. , & Schoof, C. (2018). Channelized, distributed, and disconnected: Subglacial drainage under a valley Glacier in the Yukon. The Cryosphere, 12(8), 2609–2636. 10.5194/tc-12-2609-2018

[grl64469-bib-0033] Ritz, C. , Edwards, T. L. , Durand, G. , Payne, A. J. , Peyaud, V. , & Hindmarsh, R. C. A. (2015). Potential sea‐level rise from Antarctic ice‐sheet instability constrained by observations. Nature, 528(7580), 115–118. 10.1038/nature16147 26580020

[grl64469-bib-0034] Ruina, A. (1983). Slip instability and state variable friction laws. Journal of Geophysical Research: Solid Earth, 88(B12), 10359–10370. 10.1029/JB088iB12p10359

[grl64469-bib-0035] Schoof, C. (2005). The effect of cavitation on glacier sliding. Proceedings of the Royal Society A: Mathematical, Physical and Engineering Science, 461(2055), 609–627. 10.1098/rspa.2004.1350

[grl64469-bib-0036] Schoof, C. (2010). Ice‐sheet acceleration driven by melt supply variability. Nature, 468(7325), 803–806. 10.1038/nature09618 21150994

[grl64469-bib-0037] Schoof, C. , Hewitt, I. J. , & Werder, M. A. (2012). Flotation and free surface flow in a model for subglacial drainage. Part 1. Distributed drainage. Journal of Fluid Mechanics, 702, 126–156. 10.1017/jfm.2012.165

[grl64469-bib-0038] Shepherd, A. , Ivins, E. , Rignot, E. , Smith, B. , van den Broeke, M. R. , Velicogna, I. , et al. (2019). Mass balance of the Greenland Ice Sheet from 1992 to 2018. Nature, 579, 233–239. 10.1038/s41586-019-1855-2 31822019

[grl64469-bib-0039] Shreve, R. L. (1972). Movement of water in glaciers. Journal of Glaciology, 11(62), 205–214. 10.3189/S002214300002219X

[grl64469-bib-0040] Sommers, A. , Rajaram, H. , & Morlighem, M. (2018). SHAKTI: Subglacial hydrology and kinetic, transient interactions v1.0. Geoscientific Model Development, 11(7), 2955–2974. 10.5194/gmd-11-2955-2018

[grl64469-bib-0041] Thøgersen, K. , Gilbert, A. , Schuler, T. V. , & Malthe‐Sørenssen, A. (2019). Rate‐and‐state friction explains glacier surge propagation. Nature Communications, 10(1), 2823. 10.1038/s41467-019-10506-4 PMC6597537 31249287

[grl64469-bib-0042] Tsai, V. C. , Smith, L. C. , Gardner, A. S. , & Seroussi, H. (2021). A unified model for transient subglacial water pressure and basal sliding. Journal of Glaciology, 68, 1–400. 10.1017/jog.2021.103

[grl64469-bib-0043] Vincent, C. , & Moreau, L. (2016). Sliding velocity fluctuations and subglacial hydrology over the last two decades on Argentière glacier, Mont Blanc area. Journal of Glaciology, 62(235), 805–815. 10.1017/jog.2016.35

[grl64469-bib-0044] Vincent, C. , Soruco, A. , Six, D. , & Meur, E. L. (2009). Glacier thickening and decay analysis from 50 years of glaciological observations performed on Glacier d’Argentière, Mont Blanc area, France. Annals of Glaciology, 50(50), 73–79. 10.3189/172756409787769500

[grl64469-bib-0045] Walder, J. S. (1986). Hydraulics of subglacial cavities. Journal of Glaciology, 32(112), 439–445. 10.3189/S0022143000012156

[grl64469-bib-0046] Walder, J. S. , & Fowler, A. (1994). Channelized subglacial drainage over a deformable bed. Journal of Glaciology, 40(134), 3–15. 10.3189/S0022143000003750

[grl64469-bib-0047] Werder, M. A. , Hewitt, I. J. , Schoof, C. G. , & Flowers, G. E. (2013). Modeling channelized and distributed subglacial drainage in two dimensions. Journal of Geophysical Research: Earth Surface, 118(4), 2140–2158. 10.1002/jgrf.20146

[grl64469-bib-0048] Zoet, L. K. , Iverson, N. R. , Andrews, L. , & Helanow, C. (2021). Transient evolution of basal drag during glacier slip. Journal of Glaciology, 1–10. 10.1017/jog.2021.131

[grl64469-bib-0049] Chen, Y. , Liu, X. , Gulley, J. D. , & Mankoff, K. D. (2018). Subglacial conduit roughness: Insights from computational fluid dynamics models. Geophysical Research Letters, 45(20), 206–218. 10.1029/2018GL079590

[grl64469-bib-0050] Hewitt, I. J. , Schoof, C. , & Werder, M. A. (2012). Flotation and free surface flow in a model for subglacial drainage. Part 2. Channel flow. Journal of Fluid Mechanics, 702, 157–187. 10.1017/jfm.2012.166

[grl64469-bib-0051] Vincent, C. (2002). Influence of climate change over the 20th Century on four French glacier mass balances. Journal of Geophysical Research: Atmospheres, 107(D19), 1–12. 10.1029/2001JD000832

