# Supplementary material for: A Consistent Framework for Coupling Basal Friction With Subglacial Hydrology on Hard‐Bedded Glaciers
Source: Geophys Res Lett. 2022 Jul 8;49(13):e2021GL097507. doi: 10.1029/2021GL097507 (PMC9540583; doi:10.1029/2021GL097507)
Supplement: Supplementary file 1 — Supporting Information S1 [file GRL-49-e2021GL097507-s001.pdf]

AGU PUBLICATIONS

Geophysical Research Letters

Supporting Information for

A Consistent Framework for Coupling Basal Friction with Subglacial Hydrology on Hard-bedded Glaciers

Adrien Gilbert[1,2], Florent Gimbert[1], Kjetil Thøgersen[3], Thomas V. Schuler[2] and Andreas Kääb[2]

[1]CNRS, IRD, Grenoble INP, IGE, University Grenoble-Alpes, CS 40700, 38 058 Grenoble Cedex 9, France.
[2]Department of Geosciences, University of Oslo, Boks 1072 Blindern, NO-0316 OSLO, Norway.
[3]Physics of Geological Processes, The Njord Centre, Department of Physics, University of Oslo, Boks 1072 Blindern, NO-0316 OSLO, Norway.

Contents of this file

Text S1 to S4
Figures S1 to S8
Tables S1 to S2

Introduction

In this supporting information we provide further details on the model equations and set up (**Text S1**), on the estimation of parameters (**Text S2**) and on the comparison of our model predictions with previous approaches (**Text S3** and **S4**). This supporting information also includes additional figures and tables that are referenced in the main text.

Text S1. Model description

1. <u>Ice flow model</u>

The model solves the Stokes equation for temperate ice adopting Glen's flow law for viscous isotropic ice:

$$div(\boldsymbol{\sigma}) + \rho_{ice} g = 0 \qquad \text{(s1)}$$

where $\boldsymbol{\sigma}$ is the Cauchy stress tensor (MPa), g is the gravity acceleration (m s^{-2}) and ρ_{ice} is the ice density (kg m^{-3}). Basal boundary conditions are determined by the friction law described in Eq. (1) and Eq. (7) of the main text while a stress-free condition is assumed at the surface.

2. <u>Subglacial hydrology model</u>

The subglacial hydrology model mainly uses the implementation of the GlaDS model (Werder et al., 2013) in Elmer/Ice (Gagliardini & Werder, 2018) but integrates the cavitation state θ to estimate the characteristics of the distributed drainage system at any given time. Distributed (linked cavities) and channelized water flows are coupled together in order to compute the hydraulic potential Φ (MPa) at the glacier bed defined as $\Phi = \Phi_m + p_w$ with $\Phi_m = \rho_w g z_b$ the elevation potential at the glacier bed and p_w the water pressure. Flow within the distributed drainage network follows the following governing equations:

$$\frac{e_v}{\rho_w g}\frac{\partial \Phi}{\partial t} - \nabla \cdot \boldsymbol{Q_s} + \frac{\partial h}{\partial t} = m_{input} \quad \text{(s2)}$$

$$\frac{\partial h}{\partial t} = h_r p_1 \theta^{p_1 - 1}\frac{d\theta}{dt} \qquad \text{(s3)}$$

$$\boldsymbol{Q_s} = k_s^0 \theta^{p_2}(h_r \theta^{p_1})^{\alpha_s} \|\boldsymbol{\nabla\Phi}\|^{\beta_s - 2}\boldsymbol{\nabla\Phi} \qquad \text{(s4)}$$

where $\boldsymbol{Q_s}$ is the water sheet discharge (m^2 s^{-1}), k_s^o is the sheet conductivity, (α_s, β_s) are constant exponents, m_{input} is the water input (m s^{-1}), e_v is the englacial void ratio, ρ_w is the water density (kg m^{-3}) and (p_1, p_2) are constant exponents linking the cavitation state to the sheet thickness h as $h = h_r \theta^{p_1}$ and to the hydraulic conductivity k_s as $k_s = k_s^0 \theta^{p_2}$. Flow within channels is solved on the element edges and remain the same as described in Gagliardini & Werder (2018). Φ is set to be equal to Φ_m at the glacier front, and a no input flux condition is applied at the glacier outline boundary.

3. <u>Coupling ice flow and subglacial hydrology</u>

Following a previous implementation of GlaDS in Elmer/Ice (Gagliardini & Werder, 2018), the hydrological model is solved within the finite element code Elmer/Ice on a 2D mesh at the glacier base, while the Stokes equation is solved in 3D on the whole mesh. At any given

time, the hydrological problem is first solved for a given sliding velocity in order to provide a solution for the hydraulic potential Φ and cavitation state θ. The cavitation state is then used to solve the Stokes equation and to update the sliding velocity. In order to limit the total computing time, we consider a smaller time step for solving hydrology than that for solving ice flow, given that hydraulic potential variations are much faster than the cavitation state evolution which drives the change in ice flow. We therefore use a time-step of 0.8 hour for the hydrology and 2 hours for the ice dynamics. By construction, the cavitation state results from an averaged state of effective pressure at the timescale for which the cavities evolve (several hours). There is thus no need to average the hydrological condition when driving the ice flow model at a larger time-steps as we do. We also find out that using a bigger time-step of up to 5 days for the ice dynamics does not affect the results, since the evolution of θ is not sensitive to short term velocity variations but mainly to multi-day averaged velocities. In order to further limit the computation time when doing for parameters estimation (Text S3), there we used a 100-hours' time-step for the ice dynamics.

4. External forcings: using a simplified glacier geometry and water flux description

As described in the main text, we adopt a simplified geometry represented by an 0.6 X 5 km ice slab over a bedrock with uniform slope (6° slope, Figure S1). The surface topography is constructed using the "plastic approximation" which consists in ensuring uniform driving stress and thus constructing the surface elevation by solving (Cuffey & Paterson, 2010):

$$\rho_{ice} g H \; (\sin\beta + \tan(\alpha - \beta)) = \tau_b \qquad \text{(s5)}$$

where H is the glacier thickness (m), α the surface slope and β the bed slope. Eq. (s5) can be rewritten in the form:

$$\tan\alpha = \frac{dH}{dx} - \tan\,\beta = \frac{-\tau_b + \rho_{ice} g H (\sin\beta - \tan\beta)}{\tau_b \tan\beta - \rho_{ice} g H (1 + \sin\beta\tan\beta)} \qquad \text{(s6)}$$

which allows to compute numerically the elevation of the surface z_s starting from $H = 10$ m at the glacier front (Figure S1). The temporal evolution of the surface is then imposed at daily time scale by prescribing the temporal evolution of basal shear stress reconstructed in Gimbert et al. (2021) at the location of the sliding speed measurements on the Argentière Glacier.

The coupled problem is then solved on the given evolving geometry and is forced by a time-dependent water source term m_{input} (see Eq. (s2)) prescribed at the glacier base as a function of surface elevation. We assume the source term to be equal to the surface melting (m w. eq. s^{-1}) which is computed with a degree day model forced by air temperature time series and calibrated in order to have a modeled water outlet discharge being in agreement with that observed. We use daily maximum and minimum air temperatures measured at the Lyon-Bron meteorological station applying a 6.5 K km^{-1} lapse rate to estimate the Argentière Glacier air

temperatures (Vincent, 2002). Sub-daily temperatures are computed by a sinusoidal function scaled by maximum and minimum air temperature.

Text S2. Model parameter estimation

The model parameters are summarized in Table S1 with the tuned parameters being highlighted in red. α_s , β_s, α_c, β_c, e_v are taken from the literature (Gagliardini & Werder, 2018; Werder et al., 2013) and C, h_r, l_c are fixed to realistic values. The other parameters (A_s, m, q, k_s^o, p_1, p_2, k_c, A_c) have been calibrated to minimize the mis-fit between the model and the sliding speed record of Argentière Glacier from 2000 to 2018. The model outputs are extracted and averaged along a cross section located at 1500 m from the glacier front, which is not affected by the inflow boundary condition when solving the Stokes equation.
The likelihood P of the tuned parameters is computed from the root mean square error (RMSE) between the modeled and measured sliding velocities during the period 2000-2018 assuming Gaussian distribution:

$$P = \exp\left(-\frac{1}{2}\frac{RMSE}{\sigma_d}\right). \quad \text{(S9)}$$

The standard deviation on the RMSE σ_d is fixed to 0.3 cm d^{-1}.

In winter, when no surface melting occurs, we assume that glacier sliding follows the Weertman sliding law with:

$$A_s \tau_b^m = u_b \qquad \text{(S10)}$$

where A_s is the Weertman sliding parameter (m s^{-1} Pa^{-m}) and m the Weertman sliding exponent. We first constrain A_s and m using sliding velocity and basal shear stress as measured in winter when sliding velocity is minimum (using a one-month averaged value centered on the minimum seasonal velocity each year). We find the best accordance with data for As = 6.8083·10^{-26} [5.6 – 8.4] ·10^{-26} m s^{-1} Pa$^{-3.72}$ and m = 3.72 ± 0.24 using the MATLAB® Curve Fitting toolbox (Figure S7). In the following, these values are fixed and prescribed in the coupled model prior to calibrate the other parameters.

We find that the summer speed-up follows the theoretical relationship demonstrated in this study between discharge and cavitation state, which suggests the channel system plays a minor role during the melting period at the Argentière Glacier (Figure 2). The calibration of the channel parameters (A_c,k_c) appears to be relatively independent of that of the cavity network (q, k_s^0, p_1, p_2). We therefore first find the best parameters for the channels network with fixed values of (q, k_s^0, p_1, p_2) that gives reasonable agreement between model and data in the early melting season. Using the whole sliding velocity time series (2000-2018), we find a best match between model predictions and observations for A_c = 6.0 · 10^{-24} Pa^{-3} s and k_c = 3.4 · 10^{-4} m$^{3/2}$ kg$^{-1/2}$ (Figure S8). The inferred value of k_c is several orders of magnitude lower than that proposed in the literature (Chen et al., 2018; Hewitt et al., 2012; Werder et al., 2013), which may be an artefact of the simplified geometry we use or due to an inaccurate formulation of the channel opening term in R-channel theories. For instance, the hypothesis that energy potential losses are purely converted in channel melting (Werder et al., 2013) may not be apply for grounded channels for which energy loss can also occur at the water-rock bed interface and thus not

contribute to melt. It appears that the best values of (A_c, k_c) are the ones that do not allow channels to develop significantly during the melting period but still allow channels to reduce the water pressure in winter when the sheet conductivity vanishes. The development of significant channels in summer systematically would lead to low sliding velocities in August/September that are in disagreement with the data. However, channels are still needed to explain the sliding velocity in winter as they allow to decrease water pressure and sliding speed during this period in better agreement with the data. In winter, channels exist as a dense network of small conduits and can be viewed as melt-driven opened conduits that maintain connections between cavities when mechanical, cavitation-driven opening is not sufficient.

For fixed values of A_c and k_c, we then explore the parameter space (q, k_s^0, p_1, p_2) by accounting for the fact that the values of p_1 and p_2 are constrained by the observed relationship between discharge and sliding speed, which gives $\alpha_s p_1 + p_2 = 7.2$ (Figure 2a). We thus only optimize the value of (p_1, k_s^o) for different values of q. We find that $q = 1.5$ best explains the data (Figure S2a) and that the value of q is not affecting the best values of the couple (p_1, k_s^o). We obtain p_1=0.6 and k_s^o = 0.1 m$^{7/4}$ kg$^{-1/2}$ (Figure 2a) leading to p_2=6.5. The low value of p_1 and the high value of p_2 reflect the fact that cavities open quite fast but remain unconnected until a certain threshold close to the limit of stability (transition strengthening/weakening) is reached (Figure S2b). We note that the value of C does not affect the results and only has the consequence of shifting the effective pressure in a way that the quantity $C*N$ is conserved.

Text S3. Details on Eq. (13) of the main manuscript

We combine equations (2.4), (2.5) and (2.10) of Schoof et al. (2012) with $n = m$, $k = k_s$, $q = Q_s$, $\alpha = \alpha_s$ and $\beta = \beta_s$ to derive a steady state relationship between the distributed discharge, the effective pressure and the sliding velocity. Equation (2.10) gives:

$$h = \left(\frac{\|\mathbf{Q}_s\|}{k_s \|\nabla\Phi\|^{\beta_s - 1}}\right)^{1/\alpha_s} \quad . \quad (s7)$$

Equations (2.4) and (2.5) in steady state give:

$$u_b(h_r - h) = l_r \tilde{A} h N^m$$

$$\Leftrightarrow h_r = h\left(\frac{l_r \tilde{A}}{C^m A_s}\frac{1}{\chi} + 1\right) \text{ with } \chi = \frac{u_b}{C^m N^m A_s} \quad . \quad (s8)$$

Gagliardini et al. (2007) friction law gives (see Eq. (2) of the manuscript):

$$\chi = \frac{1}{\alpha^{1/q}}\left(\frac{u_b}{A_s \tau_b^m} - 1\right)^{1/q}, \quad (s9)$$

and combined with Eq. (s8), it gives:

$$\frac{u_b}{A_s \tau_b^m} = 1 + \alpha \left(\frac{l_r \tilde{A}}{C^m A_s} \left(\frac{h}{h_r - h} \right) \right)^q . \qquad \text{(S10)}$$

Introducing the expression of *h* from Eq. (S7) gives Eq. (13) of the manuscript:

$$\frac{u_b}{A_s \tau_b^m} = 1 + \alpha \left(\frac{l_r \tilde{A}}{C^m A_s} \right)^q \frac{\|\boldsymbol{Q_s}\|^{q/\alpha_s}}{\left(h_r k_s^{1/\alpha_s} \|\boldsymbol{\nabla \Phi}\|^{(\beta_s - 1)/\alpha_s} - \|\boldsymbol{Q_s}\|^{1/\alpha_s} \right)^q} \quad . \qquad \text{(S11)}$$

Text S4. Comparison with the original Elmer/Ice – GlaDS coupled model (Gagliardini and Werder, 2018)

To compare the performance of the present framework with the previously proposed ones in explaining our data, we perform simulations using the original coupling between GlaDS and Elmer/Ice (Gagliardini & Werder, 2018). In this simulation, we however keep the transient friction approach as introduced in Thøgersen et al. (2019) (with d_c=0.5 m) in order to be able to use rate weakening friction and hourly water input. Conventional steady friction laws would indeed prevent the model to converge as effective pressure often exceed the threshold of rate weakening friction (τ_b / C) when providing water input rate at hourly time step. The coupling is done in a standard way with GlaDS solving for effective pressure at a given sliding velocity and independently of the frictional state. The sliding velocity is then updated by solving the ice flow problem for a new given effective pressure. This simulation allows to test the influence of imposing the consistency between friction and hydraulic transmissivity as done in the present approach. We optimized the distributed system parameters (α_s, $\tilde{A}$ and k_s) to obtain the best fit with the sliding velocity observations. The other parameters are set to the one used in our study. Results are described in the main manuscript with reference to Figure 2 and Figure S4.

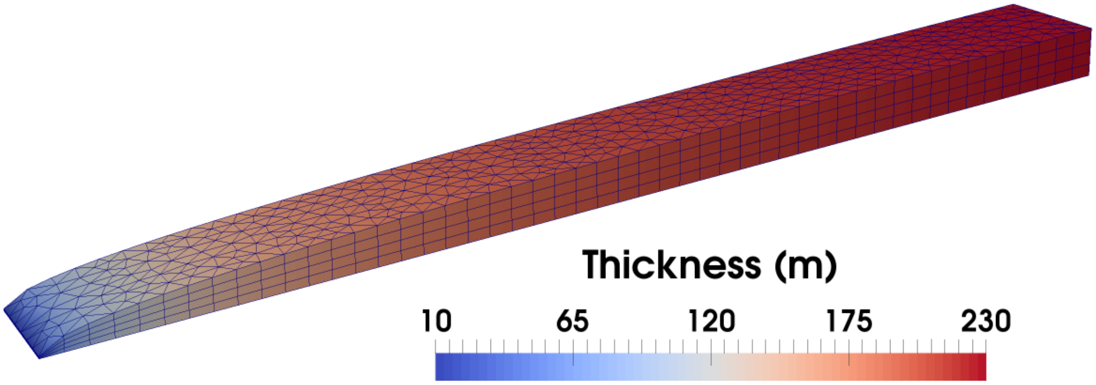

Figure S1. Idealized geometry used to solve the coupled problem. The dimensions are 600m width and 5km long for a mean bed slope of 6 degrees in order to approximate the Argentière Glacier setting.

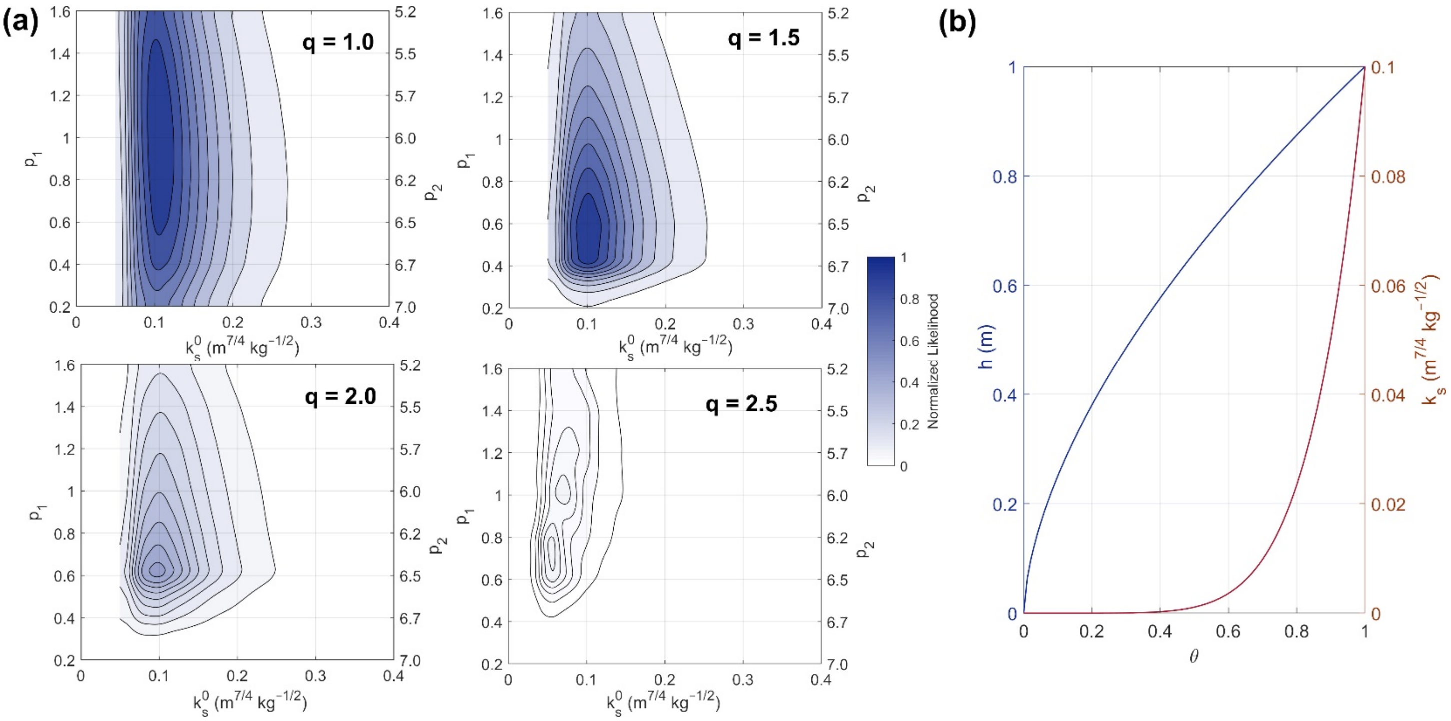

Figure S2. Parameter likelihoods computed from the misfit between modelled and measured sliding speed using the consistent hydro-mechanical coupling introduced in this study. The panels in (a) explore the parameter space (p_1, k_s^o) for different values of q and with other parameters fixed at the reference values given in Table S1. The panel (b) shows the function $h(\theta)$ and $k_s(\theta)$ obtained for the best fit parameters with q=1.5.

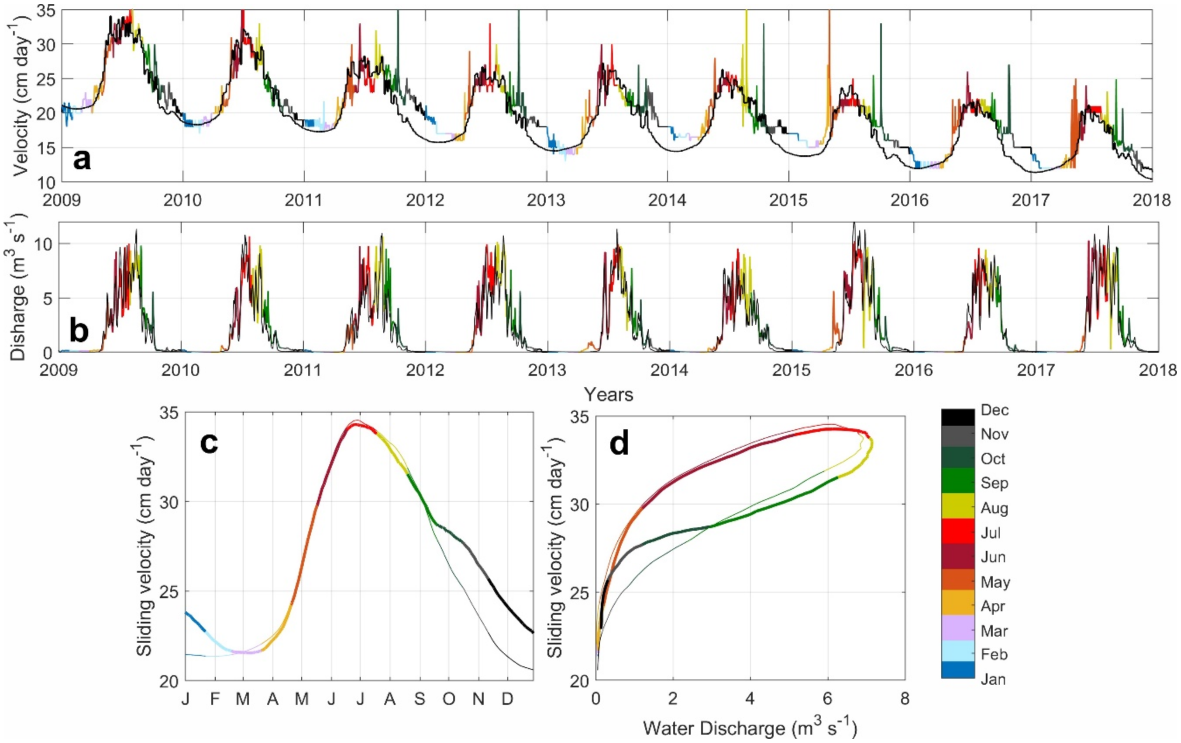

Figure S3. Comparison between modeled and observed discharge and sliding velocities using the consistent hydro-mechanical coupling introduced in this study. (a) Modelled (black line) and measured sliding velocity (color line) between 2009 and 2018. (b) Modelled (black line) and observed (coloured line) total discharge. (c) Measured (bold line) and simulated (thin line) mean sliding velocity seasonal cycle. (d) Measured (bold line) and simulated (thin line) mean cycle of sliding velocity as a function of discharge.

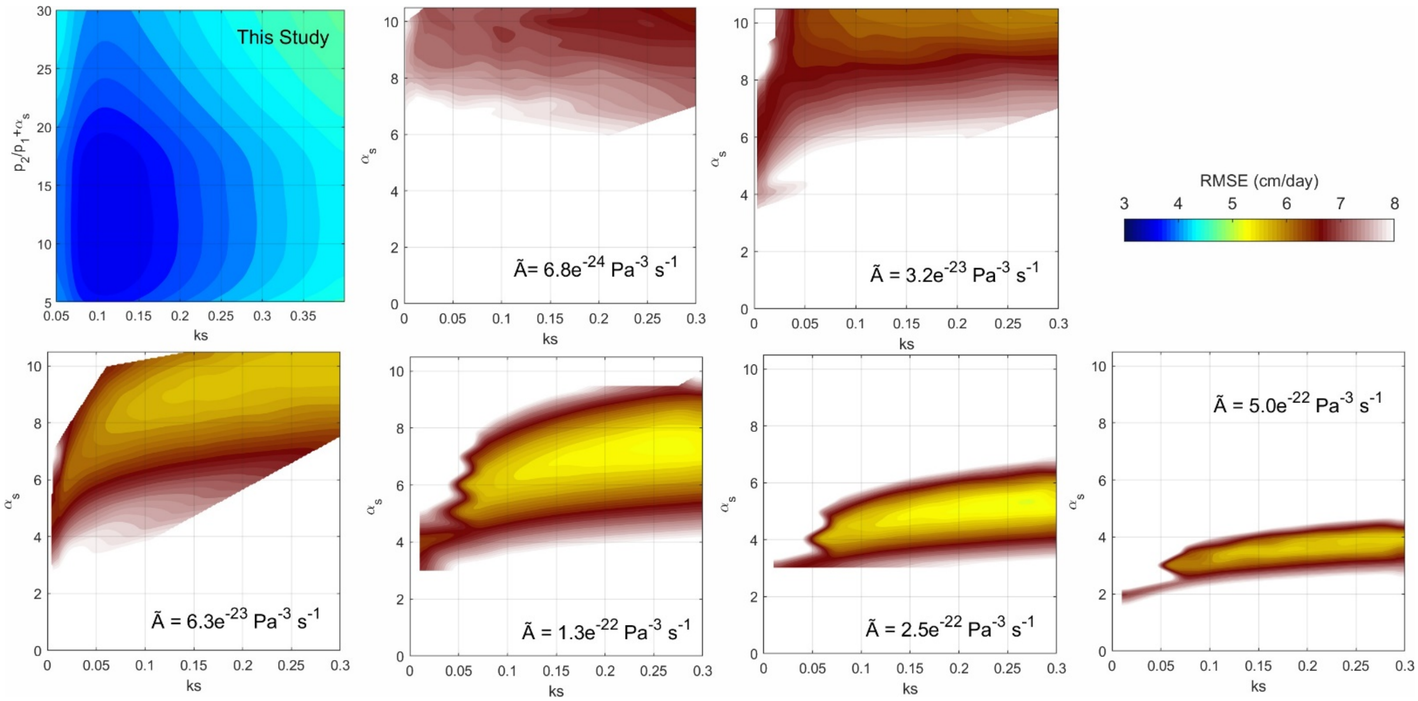

Figure S4. Root mean square error between modeled and observed sliding velocity during the period 2000-2018 using a standard coupling approach between Elmer/Ice and GlaDS (Gagliardini & Werder, 2018) for different values of the flow rate factor (closing term of cavities) compared to the error obtained using the consistent hydro-mechanical coupling (upper left panel).

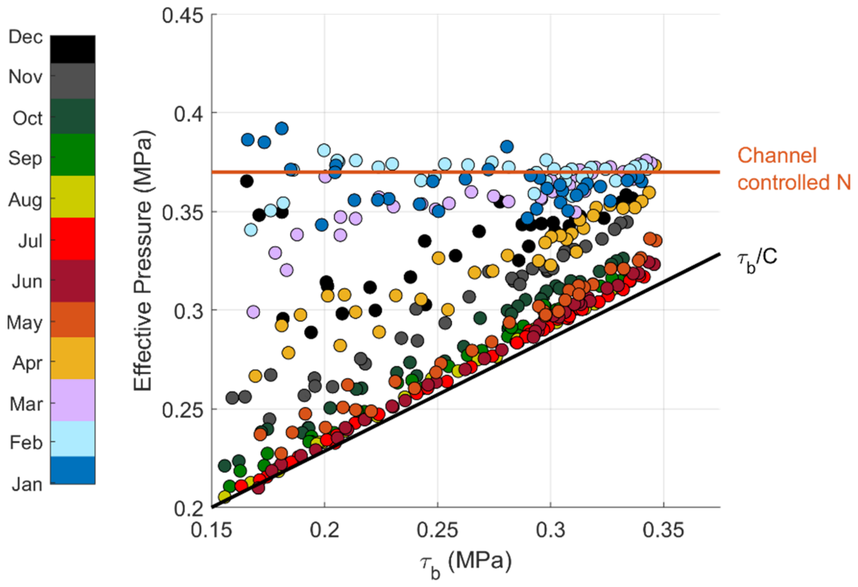

Figure S5. Monthly values of modeled effective pressure as a function of basal shear stress (dots) using the consistent hydro-mechanical coupling introduced in this study.

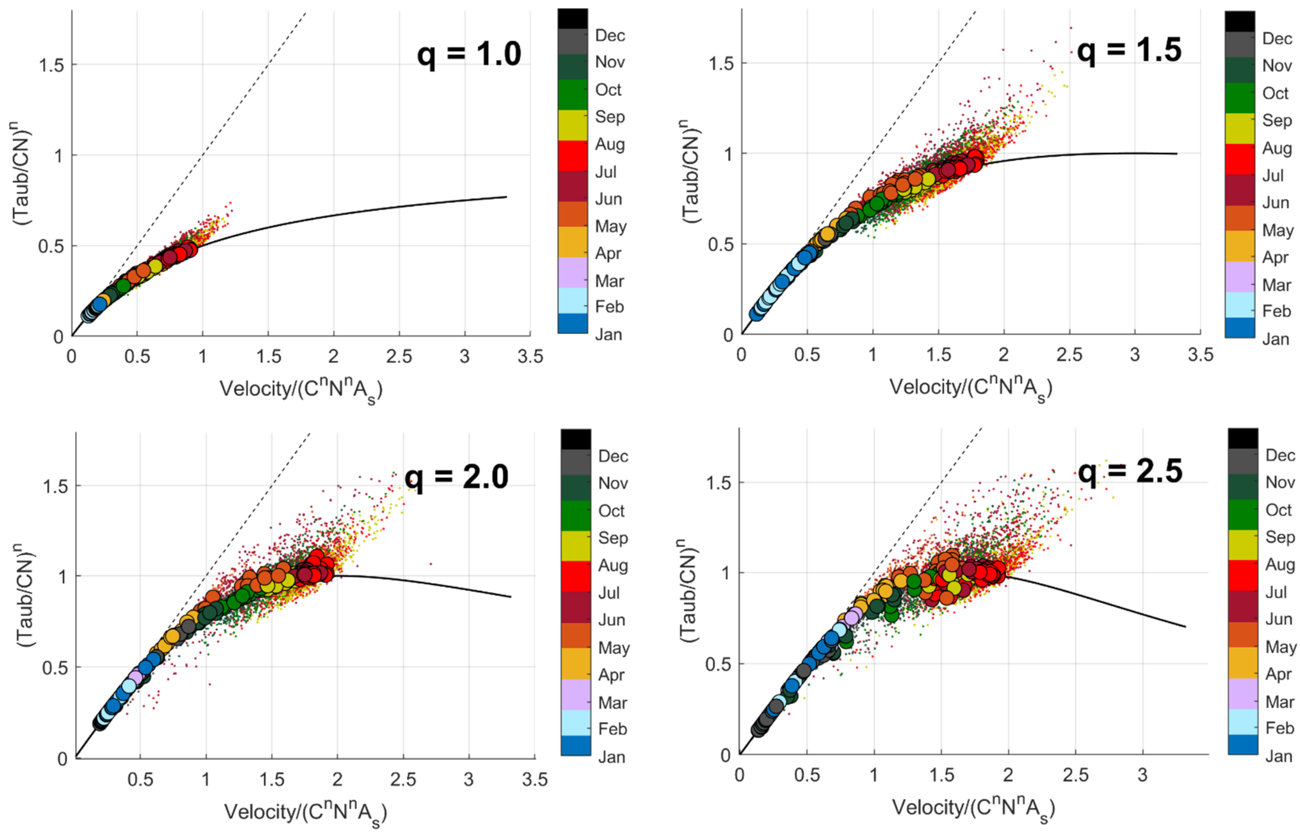

Figure S6. Modeled monthly mean values of basal shear stress, effective pressure and sliding velocity (large dots) using the consistent hydro-mechanical coupling introduced in this study compared to the steady state sliding law (black bold line) for different values of q. The dashed line represents the Weertman law. The value of k_s^0 and p_1 are fixed to 0.1 $m^{7/4}\,kg^{-1/2}$ and 0.6 respectively.

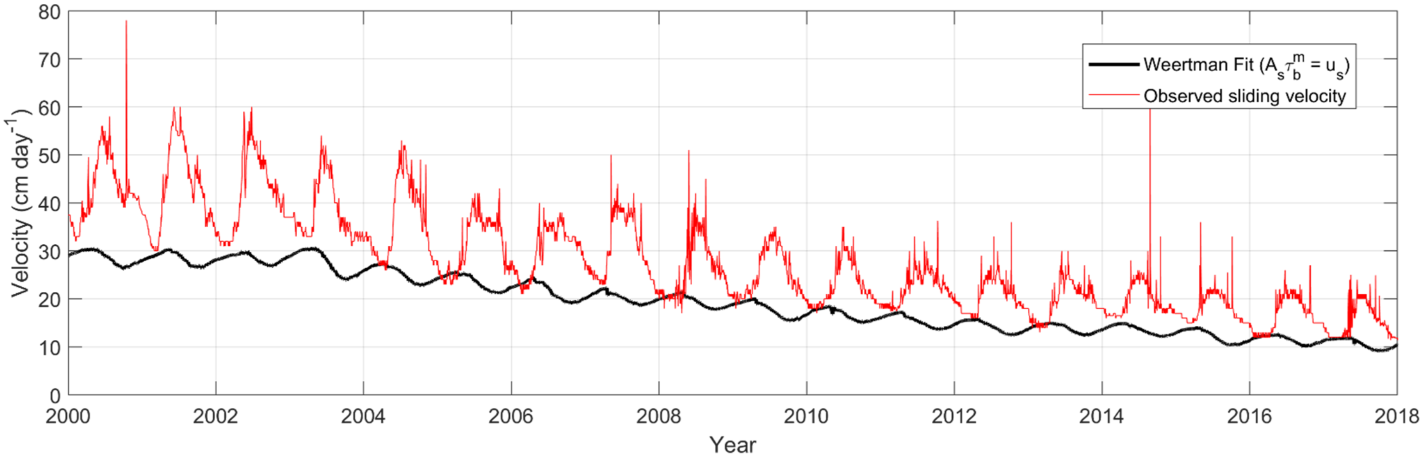

Figure S7. Observed sliding velocity at the Argentière wheel (red) and modelled sliding velocity using a Weertman law calibrated on observed minimum velocities each year. See section S2 for basal shear stress estimation used here.

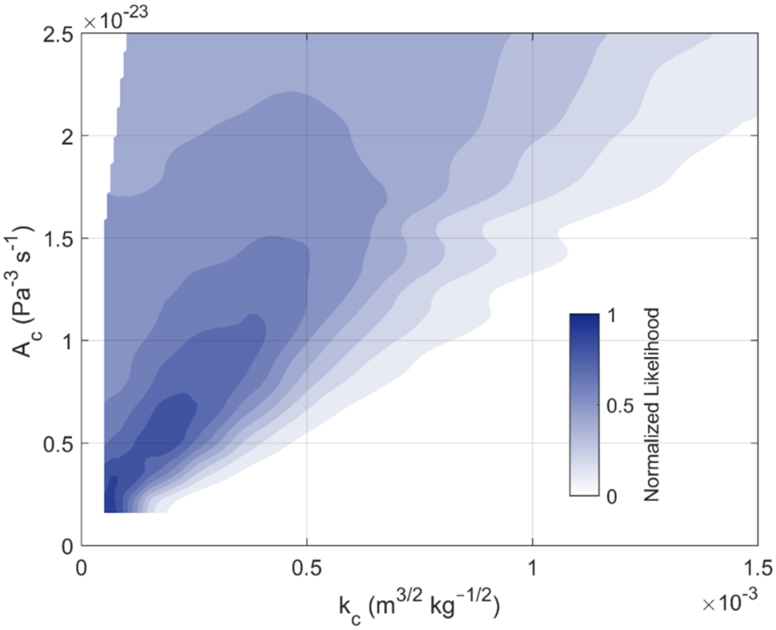

Figure S8. Channel parameters likelihood computed from the misfit between modelled and measured sliding speed using the consistent hydro-mechanical coupling introduced in this study. The figure explores the parameter space (A_c,k_c) for other parameters fixed at the reference values given in Table S1.

Table S1. Parameters of the model using the consistent hydro-mechanical coupling introduced in this study. In red the parameters which are calibrated in this study.

Description	Symbol	Values	Units
**Physical Constants**			
Gravity constant	g	9.8	$m\ s^{-2}$
Ice density	ρ_{ice}	917	$kg\ m^{-3}$
Water density	ρ_w	1000	$kg\ m^{-3}$
Latent heat	L	$3.34 \cdot 10^5$	$J\ kg^{-1}$
Clausius-Clapeyron constant	c_t	$7.5 \cdot 10^{-8}$	$K\ Pa^{-1}$
Heat capacity of water	c_w	$4.22 \cdot 10^3$	$J\ K^{-1}\ kg^{-1}$
**Ice flow model**			
Glen exponent	n	3	$J\ kg^{-1}$
Fluidity	A	$2.5 \cdot 10^{-24}$	$Pa^{-3}\ s^{-1}$
Weertman sliding parameter	A_s	$6.8083 \cdot 10^{-26}$	$m\ s^{-1}\ Pa^{-3.72}$
Iken's bound	C	0.7	
Weertman exponent	m	3.72	
Friction law shape exponent	q	1.5	
Space between bedrock bumps	l_r	2.0	m
**Hydrological model : sheet**			
Sheet flow exponent	α_s	5/4	
Sheet flow exponent	β_s	3/2	
Sheet conductivity	k^o_s	0.1	$m^{7/4}\ kg^{-1/2}$
Sheet thickness exponent	p_1	0.6	
Sheet conductivity exponent	p_2	6.5	
Bedrock bump height	h_r	1.0	m
**Hydrological model : channels**			
Channel flow exponent	α_c	5/4	
Channel flow exponent	β_c	3/2	
Channel conductivity	k_c	$3.4 \cdot 10^{-4}$	$m^{3/2}\ kg^{-1/2}$
Sheet width below channels	l_c	1.0	m
Channel creep closure coeff.	A_c	$6.0 \cdot 10^{-24}$	$Pa^{-3}\ s^{-1}$
**Hydrological model : general**			
Englacial void ratio	e_v	$8.0 \cdot 10^{-4}$	

Table S2. Variables of the model using the consistent hydro-mechanical coupling introduced in this study.

Description	Symbol	Units
Velocity	$\boldsymbol{u}$	m yr^{-1}
Stress tensor	$\boldsymbol{\sigma}$	MPa
Cavitation ratio	θ	-
Hydraulic potential	Φ	MPa
Sheet thickness	h	m
Channel cross-sectional area	S	m^2
Effective pressure	N	MPa
Water pressure	p_w	MPa
Sheet discharge	$\boldsymbol{q}$	m^2 s^{-1}
Channel discharge	Q	m^3 s^{-1}

SI References

Chen, Y., Liu, X., Gulley, J. D., & Mankoff, K. D. (2018). Subglacial conduit roughness: insights from computational fluid dynamics models. *Geophysical Research Letters*, *45*(20), 206–218. https://doi.org/10.1029/2018GL079590

Cuffey, K. M., & Paterson, W. S. B. (2010). *The physics of glaciers* (4th ed.). Amsterdam: Academic Press.

Gagliardini, O., & Werder, M. (2018). Influence of increasing surface melt over decadal timescales on land-terminating Greenland-type outlet glaciers. *Journal of Glaciology*, *64*(247), 700–710. https://doi.org/10.1017/jog.2018.59

Gimbert, F., Gilbert, A., Gagliardini, O., Vincent, C., & Moreau, L. (2021). Do Existing Theories Explain Seasonal to Multi-Decadal Changes in Glacier Basal Sliding Speed? *Geophysical Research Letters*, *48*(15), e2021GL092858. https://doi.org/10.1029/2021GL092858

Hewitt, I. J., Schoof, C., & Werder, M. A. (2012). Flotation and free surface flow in a model for subglacial drainage. Part 2. Channel flow. *Journal of Fluid Mechanics*, *702*, 157–187. https://doi.org/10.1017/jfm.2012.166

Vincent, C. (2002). Influence of climate change over the 20th Century on four French glacier mass balances. *Journal of Geophysical Research: Atmospheres*, *107*(D19), 1–12. https://doi.org/10.1029/2001JD000832

Werder, M. A., Hewitt, I. J., Schoof, C. G., & Flowers, G. E. (2013). Modeling channelized and distributed subglacial drainage in two dimensions. *Journal of Geophysical Research: Earth Surface*, *118*(4), 2140–2158. https://doi.org/10.1002/jgrf.20146
